# Risk factors analysis and risk prediction model construction of non-specific low back pain: an ambidirectional cohort study

**DOI:** 10.1186/s13018-023-03945-9

**Published:** 2023-07-29

**Authors:** Wenjie Lu, Zecheng Shen, Yunlin Chen, Xudong Hu, Chaoyue Ruan, Weihu Ma, Weiyu Jiang

**Affiliations:** 1 Department of Spinal Surgery, Ningbo Sixth Hospital, Ningbo, 315040 Zhejiang China; 2 Zhejiang University of Traditional Chinese Medicine Third Clinical Medical College, Hangzhou, 310000 Zhejiang China

**Keywords:** Non-specific low back pain, Risk factors, Prediction model, Nomogram

## Abstract

**Purpose:**

Non-specific low back pain (NLBP) is a common clinical condition that affects approximately 60–80% of adults worldwide. However, there is currently a lack of scientific prediction and evaluation systems in clinical practice. The purpose of this study was to analyze the risk factors of NLBP and construct a risk prediction model.

**Methods:**

We collected baseline data from 707 patients who met the inclusion criteria and were treated at the Sixth Hospital of Ningbo from December 2020 to December 2022. Logistic regression and LASSO regression were used to screen independent risk factors that influence the onset of NLBP and to construct a risk prediction model. The sensitivity and specificity of the model were evaluated by tenfold cross-validation, and internal validation was performed in the validation set.

**Results:**

Age, gender, BMI, education level, marital status, exercise frequency, history of low back pain, labor intensity, working posture, exposure to vibration sources, and psychological status were found to be significantly associated with the onset of NLBP. Using these 11 predictive factors, a nomogram was constructed, and the area under the ROC curve of the training set was 0.835 (95% CI 0.756–0.914), with a sensitivity of 0.771 and a specificity of 0.800. The area under the ROC curve of the validation set was 0.762 (95% CI 0.665–0.858), with a sensitivity of 0.800 and a specificity of 0.600, indicating that the predictive value of the model for the diagnosis of NLBP was high. In addition, the calibration curve showed a high degree of consistency between the predicted and actual survival probabilities.

**Conclusion:**

We have developed a preliminary predictive model for NLBP and constructed a nomogram to predict the onset of NLBP. The model demonstrated good performance and may be useful for the prevention and treatment of NLBP in clinical practice.

## Introduction

NLBP refers to lumbosacral pain and discomfort originating from the waist, without specific causes or structural factors, with or without radiating pain in the lower limbs. It is a prevalent clinical condition, with approximately 60–80% of adults reporting a history of NLBP, particularly among those under the age of 45 [1, 2]. While modern medical research has identified numerous complex factors contributing to NLBP, there is no clear understanding of the pathological anatomy underlying these abnormal changes. Currently, there exist diverse clinical treatment methods for NLBP. For instance, Filippo Migliorini and Alice Baroncini discovered that a combination of non-steroidal anti-inflammatory drugs, acupuncture, and transcutaneous electrical nerve stimulation can effectively alleviate pain and improve disability levels in NLBP patients [3–5]. Lorenzo Giordano et al. also found that value-added therapy is a viable option for NLBP patients who have not responded to conservative treatment [6]. Moreover, Luca Miranda et al. systematically examined 303 NLBP patients and observed that mesenchymal stem cells may inhibit nociceptors, reduce catabolism, and facilitate the repair of damaged or degenerated tissue, thereby alleviating pain [7]. However, most of these treatments offer temporary relief rather than a fundamental solution, often leaving residual symptoms. As a result, patients' expectations regarding clinical outcomes are frequently unmet, significantly impacting their physical and mental well-being [8–10]. Therefore, early screening and effective prevention of NLBP have become critical concerns for healthcare professionals. In the era of personalized medicine, accurate prediction of disease occurrence and prognosis has gained increasing importance. Constructing disease risk prediction models has proven effective in reducing disease incidence as demonstrated by numerous scholars [11, 12]. Despite notable progress in the diagnosis and treatment of NLBP in China, the lack of basic epidemiological data and a scientific prediction and evaluation system hinders successful prevention and prognosis assessment of NLBP. Therefore, the objective of this study is to identify the most significant risk factors and develop a robust risk prediction model for NLBP. The primary purpose is to provide valuable assistance to clinicians and patients in enhancing the prevention and treatment strategies for NLBP.

## Evidence before this study

We searched PubMed, Medline and CSTJ for peer-reviewed, original studies published from database inception to December 2022, with the terms “non-specific low back pain”, “NLBP”, “risk factors”, and “predictive model”. It is hoped that this study can include as many risk factors as possible to improve the clinical significance of the prediction model.

## Participants and methods

### Participants

Referring to all the risk factors obtained by searching before the survey, we conducted an ambidirectional cohort study and performed a questionnaire survey on outpatients who visited the Ningbo Sixth Hospital from December 2020 to December 2022. The inclusion criteria were as follows: age between 16 and 60 years; clinical diagnosis of lumbar myofascial pain syndrome, lumbar muscle strain, lumbar transverse process syndrome, acute lumbar sprain, sacroiliac joint arthritis, and piriformis syndrome; and clear understanding of the clinical significance of this study, voluntary participation in this study, ability to actively complete the questionnaire survey, and signing of the informed consent form. The exclusion criteria were patients lower back pain caused by nerve root compression, spinal canal stenosis, or kidney disease, specific diseases such as bone tumors, ankylosing spondylitis, spinal fractures, or spinal deformities, recurrent low back pain after spinal surgery, serious internal medicine diseases such as cardiovascular, liver, kidney, or blood system diseases, poorly controlled diabetes patients, infectious disease patients, and malignant tumor or mental illness patients. The diagnostic criteria mentioned here pertain to the diagnostic criteria for NLBP as outlined in the NLBP diagnosis and treatment guidelines of the Chinese Medical Association (2018 edition) and the NLBP diagnosis and treatment guidelines of the European Spinal Association (2021 edition) [13, 14]. This study was approved by the Ethics Committee of Ningbo Sixth Hospital (Yong Liu Yi Lun Shen 2023 Lun No.18). All methods were in accordance with the Helsinki Declaration and its contemporary amendments. Written informed consent was obtained from the patient for publication of this case report and accompanying images. A copy of the written consent is available for review by the Editor-in-Chief of this journal on request.

### Methods

We designed a survey questionnaire that included baseline information about the study participants, such as general information, work-related information, and psychological status information (Appendix). The general information included age (16–30, 31–45, 46–60 years), gender, weight, height, body mass index (BMI = weight/height^2^, ≤ 18.4 kg/m^2^, 18.5–23.9 kg/m^2^, 24.0–27.9 kg/m^2^, ≥ 28 kg/m^2^), education level, marital status (unmarried, married, divorced), smoking (occasional/no, daily), alcohol consumption (< 3 times/week, ≥ 3 times/week), exercise frequency (< 2 times/week, ≥ 2 times/week, with each exercise session lasting ≥ 30 min), place of residence (urban/city/town, rural), history of previous low back pain (there was a history of low back pain without clear cause in the past 3 months), and family history of low back pain (the incidence of NLBP in family members, a large range of family members, not limited to immediate relatives such as grandparents). The work-related information included the nature of work (physical—using physical energy to complete work tasks, mental—using intelligence to complete work tasks), daily working hours, labor intensity (as defined by the "Labor Law of the People's Republic of China," classified as light/medium, heavy/very heavy [15]), work posture (fixed—maintaining similar posture for no less than half of the working hours every day, non-fixed—maintaining a similar posture for less than half of the working hours every day), and exposure to vibration sources (such as electric drill operators, riveting machine workers, drivers, etc.). Psychological status assessment was evaluated using the Chinese version of the World Health Organization Quality of Life (WHOQOL-BREF) questionnaire [16], with the total score of each item used as the final result.

Before the survey, participants received training in NLBP and relevant survey-related knowledge. Participants were then selected based on diagnostic criteria and inclusion and exclusion criteria. After obtaining informed consent, a questionnaire was distributed to patients, who completed it independently. Investigators provided clarifications if patients had any questions regarding specific items. Upon completion, the investigators collected the questionnaires, checked for errors, and ensured their validity. The verification and recording of all data were carried out independently by two researchers.

## Statistical analysis

The data were analyzed using SPSS Statistics 25.0 software. For normally distributed continuous data, *x* ± *s* was used to represent the data, and an independent sample *t*-test was used. For non-normally distributed continuous data, *M* (P25, P75) was used, and nonparametric tests were selected. Categorical data was described using *n*(%), and a chi-square test was used. The R software (version 4.2.2) was used for model construction. Logistic regression analysis was used to identify the independent risk factors for NLBP. LASSO regression analysis was then used to select the risk prediction factors, and a binary logistic regression analysis was performed to construct the NLBP risk prediction model using whether NLBP occurred as the dependent variable. The receiver operating characteristic (ROC) curve and the area under the curve (AUC) were used to evaluate the discrimination ability of the model, and the calibration plot was used to graphically evaluate the calibration of the nomogram in the training and validation cohorts. In all analyses, *P *< 0.05 was considered statistically significant.

## Results

A total of 820 questionnaires were distributed, 791 were recovered, and 707 were valid. The effective rate of the questionnaire was 89.4%. Among the survey participants, 327 were male and 380 were female, with ages ranging from 18 to 60 years old. The diagnosis of NLBP was identified in 278 cases, resulting in a prevalence rate of 39.3%.

In this study, 19 independent variables that potentially affect the occurrence of NLBP were initially screened out. Based on the principle that "the required sample size for multiple-factor analysis should be 5–10 times that of the independent variables" [17], the required sample size in this study was 95–190. A total of 707 participants were included, which consisted of 575(80%) in the training set and 132(20%) in the validation set, exceeding the minimum required sample size and meeting the statistical requirements for sample size. Statistical analysis showed no significant differences in baseline data between the two groups (*P *> 0.05) (Table 1).

**Table 1:** Comparison of baseline data between training set and validation set

Risk factors	Training set	Validation set	Statistic	*P* value
Age (16–30/31–45/46–60)	131/257/187	37/51/44	*x*^2^ = 2.171	0.338
Gender (male/female)	269/306	58/74	*x*^2^ = 0.349	0.555
Weight	61.27 ± 5.56	60.33 ± 5.21	*t* = 1.772	0.077
Height	1.71 ± 0.15	1.69 ± 0.33	*t* = 1.055	0.292
BMI (≤ 18.4/18.5–23.9/24.0–27.9/ ≥ 28)	105/138/213/119	18/30/49/35	*x*^2^ = 3.062	0.382
Education time	10.8 ± 1.9	11.6 ± 2.2	*t* = − 1.616	0.106
Marital status (unmarried/married/divorced)	221/302/52	40/79/13	*x*^2^ = 3.068	0.216
Smoking (occasional/none, daily)	413/162	92/40	*x*^2^ = 0.238	0.625
Drinking alcohol (< 3 times/week, ≥ 3 times/week)	401/174	87/45	*x*^2^ = 0.737	0.391
Exercise frequency (< 2 times/week, ≥ 2 times/week)	432/153	105/27	*x*^2^ = 1.861	0.173
Place of residence (city/town/village)	248/205/122	53/42/37	*x*^2^ = 2.891	0.236
Past history of low back pain (No/Yes)	354/221	89/43	*x*^2^ = 1.575	0.209
Family history of low back pain (No/Yes)	253/322	58/74	*x*^2^ = 1.192	0.275
Working hours	9.1 ± 1.2	8.8 ± 1.6	*t* = 1.672	0.095
Labor intensity (light/medium, heavy/extremely heavy)	414/161	93/39	*x*^2^ = 0.126	0.722
Working posture (unfixed/fixed)	243/332	49/83	*x*^2^ = 1.170	0.279
Exposure to vibration sources (No/Yes)	471/104	105/27	*x*^2^ = 0.399	0.528
Job nature (mental/physical)	351/224	80/52	*x*^2^ = 1.513	0.219
Mental state	68.92 ± 8.71	64.57 ± 7.49	*t* = 1.817	0.070

*P *< 0.05 indicates that the difference is statistically significant

### Risk factors screening and prediction model construction

**Fig. 1:**
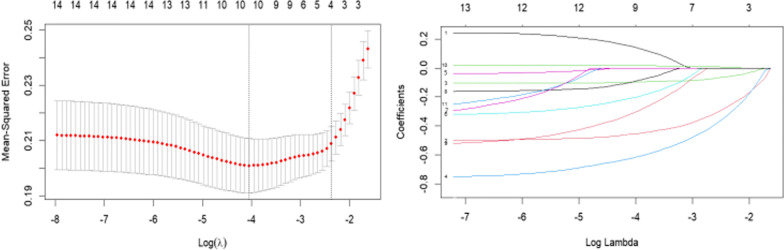
The optimal parameter selection of LASSO regression and the coefficient profile of screening independent variables. The horizontal axis X represents the logarithm of *λ*, while the vertical axis Y represents the partial likelihood error of the model. In Fig. 1, the left vertical dotted line indicates that the optimal value of lambda is approximately − 4.1, corresponding to the smallest and most accurate model prediction error, with a value of lambda.min equal to 0.01745. Conversely, the right vertical dotted line indicates that the model is simplest when the logarithm of lambda is around − 2.3, with a corresponding lambda.lse value of 0.09310

**Fig. 2:**
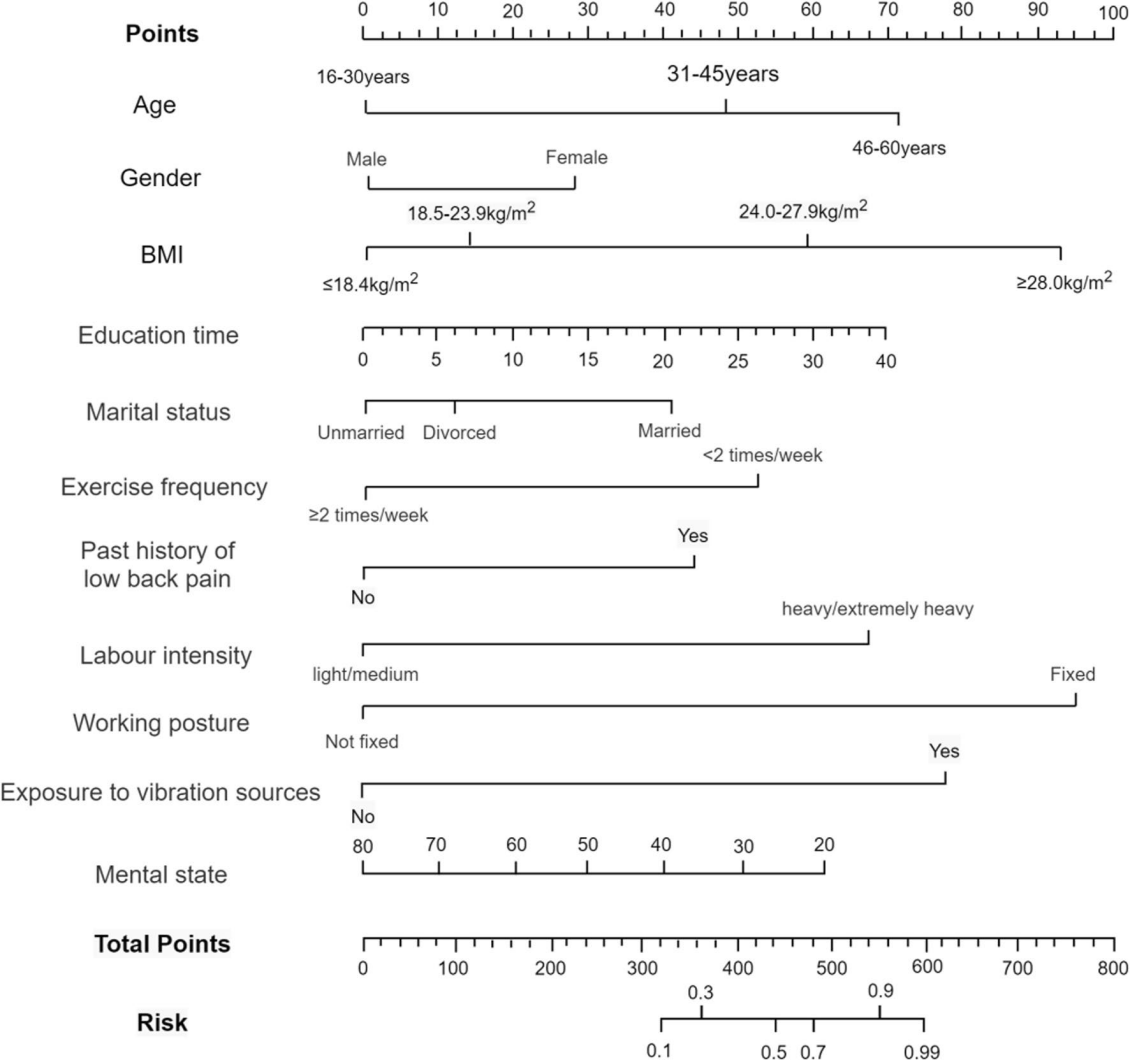
Logistic regression model nomogram. Age, gender, BMI, education time, marital status, exercise frequency, past history of low back pain, labor intensity, working posture, exposure to vibration sources, and mental state

Following single-factor, multiple-factor logistic regression, and LASSO regression analyses, a final selection of 11 predictive factors was made, including age, gender, BMI, education time, marital status, past history of low back pain, work intensity, working posture, exercise frequency, mental state and exposure to vibration sources (Fig. 1). The 11 selected predictors were included in a multivariate logistic regression model, which identified several factors that increase the risk of NLBP, including being over 30 years old, female, having a BMI of 24 kg/m^2^ or higher, having a longer education time, being married, having a past history of low back pain, engaging in heavy labor, maintaining a fixed working posture, and exposure to vibration sources. In contrast, exercising at least twice a week and having a good mental state were found to reduce the risk of NLBP. To facilitate the display of results, a nomogram was used to visualize the model (Table 2 and Fig. 2).

**Table 2:** Multivariate logistic prediction model of NLBP

Risk factors	Beta	SE	Wald	OR	95% CI	*P* value
Age	0.706	0.383	3.398	2.025	1.623 4.277	0.003
Gender	0.098	0.064	2.345	1.103	1.001 1.938	0.021
BMI	1.398	0.614	5.184	4.047	1.026 2.021	0.001
Education time	0.314	0.278	1.276	1.369	1.125 2.670	0.044
Marital status	0.318	0.560	2.372	1.374	1.013 3.181	0.025
Exercise frequency	− 0.613	0.223	5.056	0.542	0.373 0.808	0.031
Past history of low back pain	0.715	0.373	3.674	2.044	1.412 4.630	0.001
Labor intensity	1.291	0.399	7.470	3.636	1.987 5.137	0.001
Working posture	1.018	0.892	3.302	2.768	1.756 6.410	0.028
Exposure to vibration sources	1.341	0.641	4.377	3.823	1.052 5.302	0.031
Mental state	− 0.320	0.292	1.201	0.726	0.318 0.846	0.022
Constant	− 22.314	5.263	17.976	0.000	—	0.000

Coefficient value (Beta); Standard error (Beta); Chi-square value (Wald); *P* < 0.05 indicated statistical significance

The risk prediction formula for NLBP is presented as follows: In(*p*/1 − *p*) =   − 22.314 + 0.706 × 1 + 0.098 × 2 + 1.398 × 3 + 0.314 × 4 + 0.318 × 5 − 0.613 × 6 + 0.715 × 7 + 1.291 × 8 + 1.108 × 9 + 1.341 × 10 − 0.320 × 11. In this formula, × 1 represents age, × 2 represents gender, × 3 represents BMI, × 4 represents education time, × 5 represents marital status, × 6 represents exercise frequency, × 7 represents past history of low back pain, × 8 represents labor intensity, × 9 represents working posture, × 10 represents exposure to vibration sources, and × 11 represents mental state.

### Evaluation of risk prediction model

**Fig. 3:**
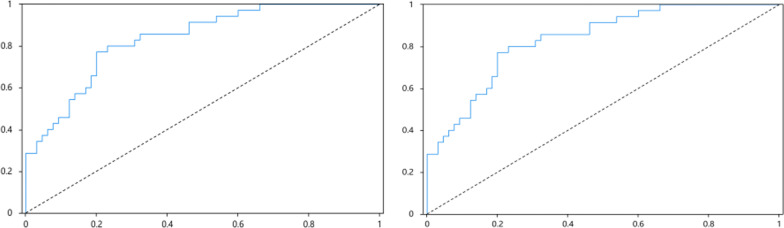
The area under the ROC curve of the training set and the validation set prediction model is visualized. The x-axis is the sensitivity, which represents the possibility of predicting positive samples but actually negative samples. The y-axis is 1-specific, which represents the possibility of predicting positive samples but actually positive samples. The blue real line is the ROC curve of the model, and the dotted line represents the ROC curve of random guess

**Fig. 4:**
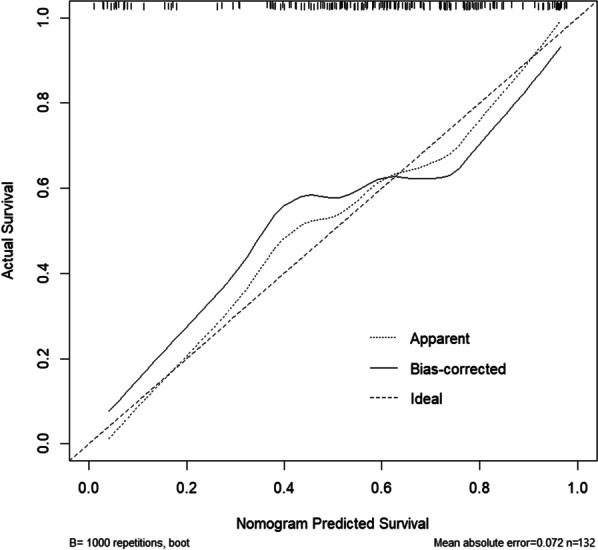
Logistic regression model HL test visualization. The x-axis represents the predicted risk of NLBP, and the y-axis represents the ratio of actual NLBP. The diagonal dotted line represents the ideal model of perfect prediction, and the solid line represents the experimental performance of the model in this study. It can be seen that the model curve is closer to the standard curve, indicating that the clinical prediction effect of the model is better

The effectiveness and applicability of the model were assessed using R software by evaluating accuracy, area under the curve (AUC) value, and stability. The validation set data were used to verify the prediction model's results. The AUC value corresponding to the predicted value in the training set was 0.835 (95% CI 0.756–0.914), and the corresponding optimal cutoff value was 0.571 (sensitivity 0.771, specificity 0.800). The AUC value corresponding to the predicted value in the validation set was 0.762 (95% CI 0.665–0.858), and the corresponding optimal cutoff value was 0.400 (sensitivity 0.800, specificity 0.600), indicating that the model had good discrimination. Additionally, a calibration curve was generated to evaluate the clinical prediction performance of the model. The x-axis represents the predicted risk of NLBP, and the y-axis represents the ratio of actual NLBP. The diagonal dotted line represents the ideal model for perfect prediction, and the solid line represents the experimental performance of the model in this study. The model curve was observed to be in close proximity to the standard curve, indicating that the clinical prediction performance of the model was superior (Table 3, Figs. 3 and 4).

**Table 3:** Logistic regression ROC results AUC summary and optimal boundary value results

Prediction value	AUC	SE	*P* value	95% CI	Best cutoff value	Sensitivity	Specificity	Cutoff
Training set	0.835	0.040	0.000	0.756 0.914	0.571	0.771	0.800	0.405
Validation set	0.762	0.049	0.000	0.665 0.858	0.400	0.800	0.600	0.513

*P*＜0.05 indicates that the difference is statistically significant

### Application example of risk prediction model

**Fig. 5:**
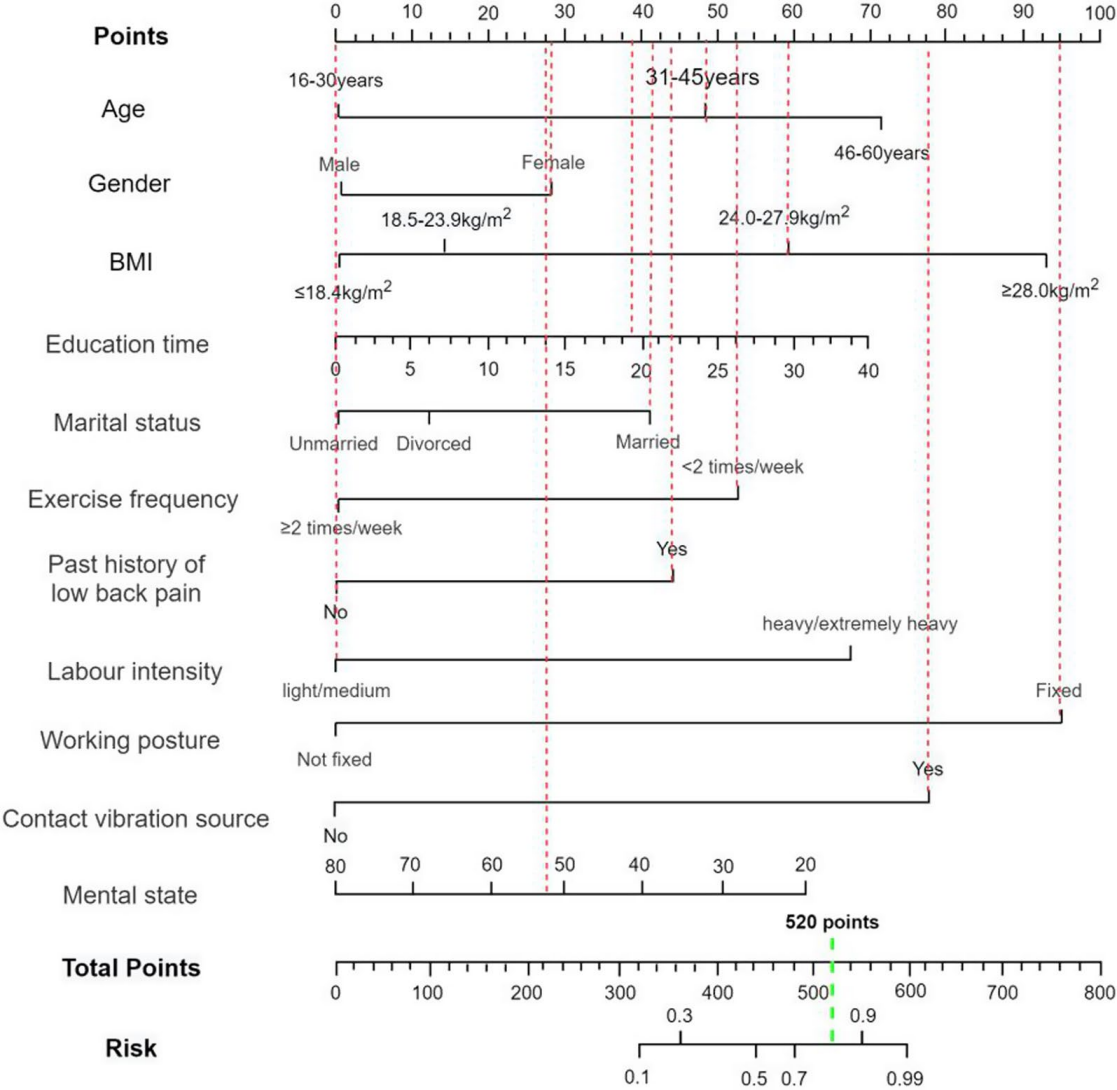
NLBP risk prediction model example. According to the data of the subjects, the corresponding scores were found in Points, which were 27 points, 58 points, 60 points, 35 points, 40 points, 52 points, 43 points, 0 points, 95 points, 80 points and 30 points respectively. The total score was 585 points. The Total Points axis was positioned at the position corresponding to 520 points, and the vertical line was downward to the Risk axis. The probability of NLBP in the patient was about 80%

To visualize the predictive model, this paper presents a nomogram that allows the derivation of the approximate probability of NLBP occurrence based on the independent variables included in the model. As an example, let us consider a female patient aged 38, who is a bus driver and her BMI is 25.8 kg/m^2^, she has 18 years of education, is married, exercises less than twice a week, has a past history of low back pain, moderate labor intensity, fixed working posture, and a WHOQOL-BREF score of 52. Based on these characteristics, the corresponding scores are obtained from the Points axis, which sum up to 520 A vertical line is then drawn downward from the position on the Total Points axis corresponding to 520 to intersect with the Risk axis. This intersection point indicates that the probability of developing NLBP in the future for this patient is approximately 80% (Fig. 5).

## Discussion

As our country has entered an aging society, the incidence of NLBP has shown a steady increase over the years. This not only affects the patients' daily lives and work but also causes great harm and economic losses to families and society. The mechanism of NLBP is still unclear, and Western medicine often has difficulty in providing effective treatment, while traditional Chinese medicine lacks specificity. Therefore, based on clinical big data and predictive medicine, effective prevention and personalized treatment of NLBP are the directions for our future research.

In line with previous studies, the incidence of NLBP in Chinese adults typically ranges between 30 and 55 years old, with the highest incidence rate occurring among those aged 41 to 50 years old. Similarly, in the USA, the most common age range for NLBP is between 30 and 60 years old, likely due to lumbar degeneration [18, 19]. Consistent with previous studies, our research has found that males have a slightly lower incidence rate of NLBP than females, possibly due to a higher prevalence of physical labor among males. However, our study also found that the incidence rate of NLBP is higher in females than males, which may be related to changes in the nature of job in society. In recent years, long working hours and fixed postures have become more common, while heavy or overweight job has decreased. This may partially explain the higher incidence rate in females. The final prediction model did not take into account the nature of job, indirectly supporting this conclusion [20]. Prolonged exposure to vibration sources can lead to muscle fatigue and damage in the lumbar region, resulting in intervertebral disk deformation and expansion, increasing pressure on lumbar nerve roots, and leading to low back pain. Damage to the intervertebral disk may cause displacement of the vertebral body, leading to instability of the lumbar spine joint, and further increasing the risk of low back pain [21]. Furthermore, a high BMI can increase the burden on the lower back's muscles, ligaments, and small joints, accelerating lumbar degeneration. The body responds by increasing the secretion of glucocorticoids, which promotes the decomposition of proteins in bones, inhibits protein synthesis, and inhibits the formation of bone matrix by bone cells [22–24]. Our research found that NLBP risk gradually increases with longer time of education, which may be due to the need for individuals with longer time of education to maintain prolonged sitting postures during work. Our study also found that physical exercise and mental state have no negative impact on NLBP and may even have a protective effect. Appropriate physical exercise can strengthen the muscles of the lower back, increase the stability of the lumbar spine, and improve the ability to withstand stress, thereby delaying the degeneration of the lumbar spine and small joints. Furthermore, a better psychological environment is conducive to reducing the incidence of NLBP, as indicated by the evaluation of the WHOQOL-BREF quality of life questionnaire. A higher score in this questionnaire indicates greater satisfaction with one's life.

The harmful effects of NLBP are well known, and current treatment strategies and methods exist only after the onset of the disease [25, 26]. Furthermore, most patients with NLBP experience varying degrees of disability and sequelae, which bring serious anxiety and economic burden to both patients and their families. Therefore, early self-screening for this disease is particularly important. In this study, a risk prediction model was adopted that visualizes the results of logistic regression based on clinical big data and predictive medicine. By integrating and coordinating the various determinants of disease occurrence, this model predicts the likelihood of a specific individual developing this clinical disease, making it easy to apply in clinical practice and meeting the needs of individual medical diagnosis and treatment. Based on the results of multiple-factor logistic regression analysis, this study found that age, gender, BMI, education level, marital status, history of low back pain, labor intensity, working posture, and exposure to vibration sources were risk factors for NLBP, while exercise frequency and psychological status were protective factors. Therefore, we constructed a risk prediction model for NLBP and validated the aforementioned model, which demonstrated a high clinical reference value with higher sensitivity and specificity.

However, the sample size included in this study was relatively small, and the ratio of training set data to validation set data was 4:1, which may have affected the predictive results due to the inconsistency between NLBP incidence rate and actual incidence rate. Future research should include larger sample sizes from multiple centers and consider adopting stricter inclusion and exclusion criteria while controlling the NLBP incidence rate to be close to the actual incidence rate. Furthermore, this study only used the logistic regression model, and in the future, multiple regression models could be compared to construct a risk prediction model with higher accuracy. Finally, it is important to acknowledge that the risk factors examined in this study were self-reported by patients, which introduces potential recall bias and subjective interpretations. In future research, efforts should be made to gather a more extensive set of clinical variables as indicators, thereby enhancing the comprehensiveness and completeness of the information captured by the predictive model. Additionally, employing a multi-center approach to sample collection would bolster the credibility of the model and strengthen its generalizability.

## Conclusion

This study presents a summary of the risk factors associated with NLBP, encompassing various variables such as age, gender, BMI, education level, marital status, history of low back pain, labor intensity, working posture, exposure to vibration sources, exercise frequency, and psychological status. Building upon these factors, a preliminary NLBP risk prediction model and nomogram were developed. The risk model demonstrates favorable discrimination and calibration, signifying its considerable clinical relevance. This model holds potential for enabling early self-screening of the condition and providing guidance for the prevention and treatment of NLBP patients.

## Data Availability

The datasets used and/or analyzed during the current study available from the corresponding author on reasonable request. Written informed consent was obtained from the patient for publication of this case report and accompanying images. A copy of the written consent is available for review by the Editor-in-Chief of this journal on request.

## Appendix

### Baseline data questionnaire

#### Mental state

**World Health Organization Quality of Life-BREF (WHOQOL-BREF)**

This questionnaire is to understand how you feel about your quality of life, health status and daily activities. Please be sure to answer all questions. If you are not sure how to answer a question, choose the answer that is closest to your true feelings. Please read each question, according to your feelings, choose the most suitable answer for your situation.
